# Leadership in Action: Exploring Nurse Leaders’ Experiences and Practices in Creating Civility: A Constructivist Grounded Theory

**DOI:** 10.1155/jonm/6944867

**Published:** 2026-03-13

**Authors:** Marianne Ota, Julia Gilbert, Louisa Lam, Danny Hills

**Affiliations:** ^1^ Institute of Health and Wellbeing, Federation University Australia, Mt Helen Campus P.O. Box 663, Ballarat, 3353, Victoria, Australia, federation.edu.au; ^2^ School of Nursing, Midwifery and Paramedicine (VIC), Australian Catholic University, 115 Victoria Parade, Fitzroy, 3000, Victoria, Australia, acu.edu.au; ^3^ School of Public Health and Preventive Medicine, Monash University, Wellington Rd., Clayton, 3800, Victoria, Australia, monash.ac.za

## Abstract

**Background:**

Civility is essential to ensuring the wellbeing of nurses and the delivery of quality patient care. Given that nurse leaders influence the behaviours and expectations of nurses in their teams within health and aged care settings, further insight is needed in understanding the practices of nurses holding leadership positions in creating civility.

**Purpose:**

The aim of this study was to explore the experiences of nurse leaders in promoting and maintaining civility in nursing care teams in Australian regional and rural health and aged care settings.

**Methods:**

Using a constructivist grounded theory methodology, 11 regional and rural nurse leaders in Victoria, Australia, were individually interviewed. Interview transcripts, recordings and memos were analysed using reflexivity and constant comparison to inform the substantive theory.

**Findings:**

The substantive theory titled *ACTS Theory for Creating Civility in Nursing* conceptualises the practices of nurse leaders in creating civility across four elements (a*cknowledge, communicate, teach* and *support*). Acknowledging individual skills and personal circumstances, practising compassionate communication, role‐modelling acceptable behaviours and providing support were core practices in fostering civility.

**Discussion:**

Ongoing implementation and evaluation of precepting, mentoring, and professional accountability programmes aimed at improving nurse leaders’ communication and leadership skills is needed to address this ongoing workforce issue.

## 1. Introduction

Civility is a positive workplace behaviour that occupies a small body of peer‐reviewed literature aimed at testing civility interventions or investigating ethical leadership styles. A term often interchanged with “politeness”, civility is widely acknowledged as the act of respecting others regardless of one’s beliefs or opinions [1]. With strong ethical implications in nursing practice, Hudson [2] argues that civility is the conscious act of honouring the dignity of another person, a virtue reflected in nursing codes of ethics worldwide [3, 4].

While evidence suggests that civility is beneficial to nurses’ mental and physical health, resulting in reduced burnout and less absenteeism [5], unprofessional behaviours are closely linked to workplace incivility. Opposite to civility, workplace incivility is a negative workplace behaviour that occurs when nurses display disrespectful behaviours to one another, such as when gossiping, complaining or making sarcastic or intimidating remarks [6].

Nurses experience higher levels of workplace incivility than any other health professional, with forms of verbal abuse accounting for the majority of uncivil behaviours [7]. In Australia, 49% of nurses reported experiencing workplace incivility within the preceding month [8]. The frequency of workplace incivility in nursing is not only alarming but also has far‐reaching effects within the profession. Workplace incivility not only compromises team and clinical performance [9] but also the health and wellbeing of nurses [10] and the quality of patient care [11, 12]. Widespread behaviours of disrespect between nurses are therefore a concerning and ongoing workforce issue.

Within healthcare organisations, nurse leaders largely determine the acceptable behaviours, expectations and culture of nurses in nursing care teams [13]. Nurse leaders are ideally placed to address workplace incivility and promote and maintain civility amongst all nurses. A recent scoping review of the literature regarding nurse leaders’ practices in fostering civility [14] revealed the scarcity of qualitative research regarding this phenomenon. The majority of previous research has applied quantitative methods to investigate either the associations between civility and ethical leadership styles [13, 15–17] or to test a civility intervention [18, 19]. A methodological gap therefore exists in understanding this phenomenon through a qualitative lens. There is a need for the perceived practices of nurse leaders in promoting and maintaining civility to be further clarified, and for the possible barriers and challenges that impact on these practices to be identified. It is essential to understand how nurse leaders pragmatically promote and maintain civility in nursing care teams to inform future nursing management practices.

The overall aim of this study was to explore the experiences of nurse leaders in promoting and maintaining civility in Australian regional and rural health and aged care settings.

The following research questions were developed:

1. What practices do nurse leaders employ to promote and maintain civility in nursing care teams?
2. What are nurse leaders’ roles in promoting and supporting civility in nursing care teams?
3. What factors influence the ability of nurse leaders to seek support in promoting and maintaining civility in nursing care teams?
4. What barriers do nurse leaders face in promoting and maintaining civility in nursing care teams?

## 2. Methodology and Methods

This study employed constructivist grounded theory to achieve the research aim and answer the research questions. Constructivist grounded theory [20] is an interpretive form of grounded theory widely applied in qualitative research. While traditional forms of grounded theory position the researcher as an objective observer [21], Charmaz’s [20] approach to constructivist grounded theory acknowledges the lead researcher’s subjectivity in interpreting and constructing data through reflexivity. Reflexivity is the process of becoming aware of one’s own privileges, preconceived ideas and biases, which informs decisions regarding how to group and conceptualise categories and codes. In the current study, the lead researcher’s extensive experience of working as a nurse in a leadership position and managing uncivil behaviours in nursing care teams was therefore valuable in seeking to answer the research questions. Memoing and routine discussions with other members of the research team regarding the developing theory supported the maintenance of this reflexive stance.

The substantive theory that is produced from the analyses is the lead researcher’s interpretation formed from their shared relationships, experiences and perceptions held with the participants [20]. The substantive theory does not seek to prescribe or formulate practice step‐by‐step. Instead, it aims to generate fresh insight into a specific phenomenon, one that is co‐constructed and inherently interpretive in nature [20]. This flexibility allows for the development of a unique understanding of a given area of study that can be built upon as new insights occur [20].

Constructivist grounded theory applies a symbolic interactionist perspective by viewing interaction as a symbolic process through which unspoken and spoken language and meanings occur [22]. This perspective entails continuously creating new ways of “being” in the world, like when there has been a loss or change [20]. Given the emotional nature of workplace incivility, Loh and Saleh [23] argue that this process is relevant when studying the process of managing uncivil behaviours.

## 3. Participants

Using purposive sampling, nurse leaders from regional and rural areas of one Australian state (Victoria), who worked in the health or aged care sector, were invited to participate in this study. An interview invitation was circulated to potential participants by email and social media containing an online link. Upon accessing the link, potential participants were taken to the Qualtrics platform and asked to complete an online questionnaire, which contained the consent form, the plain language information statement (PLIS) and questions about participants’ demographics. The questionnaire also contained the lead researcher’s contact details to ensure that potential participants could ask questions regarding the study. Written informed consent was obtained from all participants.

The lead researcher contacted participants who had registered their interest in the study to organise a time and date for individual semistructured interviews by email or phone. Additional participants were recruited via the snowballing sampling method. New questions were developed as the interviews progressed, and participants were asked to recommend new participants, thereby reflecting the process of theoretical sampling [24]. Recruitment of participants ceased upon reaching theoretical saturation when the data elicited no new areas of inquiry [20].

## 4. Data Collection

Data were collected through individual semistructured interviews conducted online using Microsoft Teams. Following participants’ consent to audio and video recording, interviews were automatically transcribed, downloaded and securely stored on the university’s OneDrive. All transcripts were amended for accuracy after each interview. An interview guide provided a guide for questioning during the interviews and was routinely reviewed to reflect the developing analyses [20]. In addition to transcripts and recordings, data were collected from memos written by the lead researcher to explore developing codes, categories and the substantive theory. To ensure validity, all participants consented to their quotes being used in this paper.

## 5. Data Analysis

Data were collected and analysed simultaneously using the constant comparative method. After each interview, study data produced from the transcripts, recordings and memos were coded. Similar codes were grouped together under tentative categories. As the interviews and concurrent analyses progressed, categories across all the interviews were clustered to form tentative themes and compared with the data generated from previous interviews. Tentative themes were refined and discussed with the research team to inform the substantive theory. Consistent with the constructivist grounded theory approach [20], when the researcher observed that the data incited no new ideas, data collection ceased, thereby achieving theoretical saturation.

Quality in a constructive grounded theory study is judged by four criteria: credibility, originality, resonance and usefulness [20]. Credibility involves becoming familiar with the research topic and setting to ensure that the research has sufficient evidence for the claims made by the researchers [20]. This was achieved in the current study by ensuring that the values and stories of the participants were fully reflected in the substantive theory. This process involved continually comparing and analysing codes and categories through memo writing and discussing the content of these memos with the research team to gain further clarity and to identify assumptions and potential biases held by the researcher. The second criterion of originality, which ensures that the research provides new insights and knowledge regarding a phenomenon [20], was achieved by conducting a scoping review which identified the scarcity of knowledge regarding the practices of nurse leaders in creating civility. The study also demonstrated resonance, or the degree to which the substantive theory holds meaning to relevant people [20], by revisiting memos, transcripts and recordings and comparing new insights against the developing analysis. Lastly, the usefulness of the study, which refers to the practicality of the research [20], is reflected in the use of the acronym “ACTS” which serves as a memorable and functional way of remembering the substantive theory which may be useful in a wide variety of practical and educational settings and applications. The COREQ checklist was applied as a measure to evaluate the quality of reporting in this paper [25].

## 6. Ethical Considerations

Ethical approval was granted from the university’s Human Research Ethics Committee (Ref No. 2022‐160). Participants were informed, in the PLIS, of the voluntary nature of the study and that they could withdraw their participation at any time. The PLIS contained information outlining measures to protect participant confidentiality and privacy. For example, each participant was given a pseudonym, and the names of healthcare organisations and units were removed from transcripts to protect participant privacy. The researcher also spoke with participants about accessing appropriate support services, before and after the interviews, if discussing workplace incivility became distressing to them at any stage.

## 7. Results

A total of 11 nurse leaders from regional and rural areas of one Australian state (Victoria), who were working in the health or aged care sector, were recruited for this study. Participants ranged in age from 34 to 69 years, and all participants had previous experience working in a leadership role. Within the context of this study, any nurse who had experience managing a nursing care team to deliver care was considered a nurse leader. Within regional and rural settings in Victoria, participants reported working in a variety of healthcare settings, ranging from large, regional hospitals to smaller‐scale rural hospitals and residential aged care facilities. Most participants were employed on a part‐time basis as nurse educators in a regional hospital, with many describing working between three and 15 years in a leadership role such as a nurse educator, graduate nurse coordinator, team leader or nurse unit manager. Their clinical specialities ranged from aged care to more acute‐focused settings, including intensive care and emergency care. The geographical location, sectors and work settings from which participants reported working in reflected the nursing backgrounds and clinical expertise of the researchers undertaking this constructivist grounded theory study [20].

**FIGURE 1:**
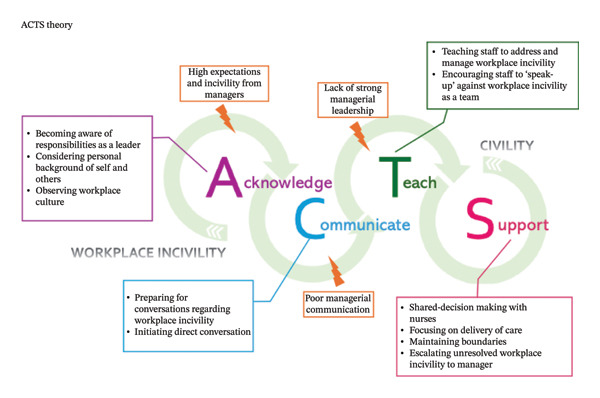
ACTS theory.

Figure 1 presents the substantive theory reflective of nurse leaders’ practices in promoting and maintaining civility in regional and rural health and aged care settings within nursing care teams. The theory contains four elements (*acknowledge, communicate, teach* and *support*) which are depicted at the top of the figure. The first letter of each element can be read both as the full word (i.e., “A” for “acknowledge”) or together as an acronym ACTS that incorporates the first letter of each element. The “ACTS” acronym was chosen to reflect the “acts” or “practices” of nurse leaders in creating civility. The curved arrows positioned horizontally link the letters of the acronym together, reflecting the consecutive process of the phenomenon. Each element is linked to a description of relevant practices and barriers within Figure 1. The barriers provide further insight into the contextual factors which impacted nurse leaders’ ability to create civility in nursing care teams. Table 1 presents the progression of codes, categories and themes that informed the theory. The following sections define and describe the interrelationships between each element of the ACTS theory.

**TABLE 1:** Development of codes and conceptual categories for each element of ACTS.

Codes	Conceptual categories	Element
Reflect on what it means to be a leader Identify own gaps in managing workplace incivility Seek out appropriate education and training regarding workplace incivility Reflect on own upbringing, and family background Consider societal or cultural implications Differing personalities Being on‐the‐floor Watching staff interactions vs High expectations from managers	Becoming aware of responsibilities Considering personal background of self and nurse Observing workplace culture	Acknowledge

Decide on appropriate time and place Establish psychologically and physically safe setting Protect privacy Consider having a witness Direct, face‐to‐face conversations Open and nonjudgemental approach Seek rationale for workplace incivility Actively listen to response Document conversation vs Poor managerial communication	Preparing for conversations Providing the message	Communicate

Build on knowledge from previous conversations Role‐model effective workplace incivility management Encourage nurses to call out uncivil behaviours Work together to address workplace incivility vs Poor managerial leadership	Teaching appropriate behaviours Managing workplace incivility together as a team	Teach

Seek clinical input Ask opinion regarding workplace decisions Meet patient or resident needs Achieve workplace goals Leave personal life at the door	Sharing decisions with nurses Focusing on delivery of care Maintaining boundaries	Support

### 7.1. Acknowledge

The first letter ‘A’ in the acronym ACTS represents the element *acknowledge* and reflects the process in which participants acknowledged their responsibilities as a leader. As the first step in creating civility in nursing care teams, this involved understanding their role as a leader, identifying any gaps in their understanding of managing workplace incivility, recognising any personal factors affecting the nurses they were leading and gathering a sense of workplace culture. The term ‘acknowledge’ is appropriate given that the traditional definition of the word is to recognise, admit or accept one’s own situation. For example, as part of understanding their role as a leader, many of the participants reflected on the benefits of creating civility for their team, and the positive impact on patients and staff on a wider scale motivated participants to address uncivil behaviours as described by Olivia below.

I think of the benefits. I was, like, “Well, if I don’t talk to the person, I’m going to be suffering mentally, emotionally, so I really have to be brave to face the situation”, so it’s for my own good and, of course, for the good of everyone.

Realising their responsibilities as leaders in creating civility led participants to seek education and training to address their own gaps in knowledge, particularly in relation to conflict resolution. However, participants often faced barriers in seeking out appropriate training to meet their learning needs. Knowledge from peers and mentors and past personal and professional experiences, instead, helped participants gain confidence in creating civility.

Acknowledging one’s background, upbringing, family situation and personality were also important practices. Participants not only acknowledged these factors within themselves but also considered how they affected other nurses’ ability to resolve workplace incivility within their team. Participants acknowledged a number of personal circumstances within nurses, including culture, age, personality and emotional status. Spending time observing how nurses treated one another in the clinical environment was also an important practice to gather a sense of workplace culture. Anna revealed:

That’s my biggest secret … Sometimes I would do that medication round and I wouldn’t be the unit manager behind the drug trolley. No one would see me, so I could hear lots of things. Staff grumblings … two nurses huddled in the corner talking about something, someone running around trying their hardest … They’re just examples that I could address there and then … Sort of like a fly on the wall.

While participants gained confidence through the practices described above in creating civility, they faced some barriers with regard to their own managers. Participants described that their managers sometimes spoke disrespectfully to them or held “unrealistic” expectations regarding their skills as a leader.

The other side of the coin [is] that our leaders are rude and inappropriate or have unrealistic expectations or they’re very quick to say, “You’re not good enough” … To me, I think that’s a useless piece of information … And I think that’s something that I’ve noticed particularly increasing.

### 7.2. Communicate

The second element of the ACTS theory represents the element of *communicate*, wherein, having acknowledged a number of contextual factors in creating civility, participants described speaking directly with nurses involved in an incident of workplace incivility. As described in the previous element *acknowledge,* a period of reflection was essential prior to planning and commencing face‐to‐face conversations to ensure effective conflict resolution. The element of *communicate* is appropriate following *acknowledge* to reflect the process of seeking clarification and resolution between nurses involved in an incident of workplace incivility.

Choosing an appropriate time and place that maintained the privacy of the nurses was an important part of this process before commencing conversations. Sam stressed how this practice was essential in maintaining trust and respect with the nurses she was leading.

I’m very careful about confidentiality, and when I do have these discussions, they don’t get discussed with anyone else, apart from the people that need to be involved. I think that’s really important, and I think that builds respect as well with the people that you’re actually managing. They don’t want to hear later that you’ve bad mouthed them down the corridor.

Some participants spoke about having a witness for conversations or documenting the content of conversations for evidence. All participants commented that initiating direct conversations was challenging but essential to resolving workplace incivility. Conversations were approached in a nonjudgemental and positive manner and sought to understand nurses’ behaviours and reactions to an incident of workplace incivility. Allowing space for nurses to speak and feel “heard” was also an important practice. Georgia commented on the value of listening:

As a [nurse] in‐charge, it’s really important that you learn how to listen and listen down because it’s a lot easier for us to listen than it is for someone who feels intimidated by the hierarchy to speak up.

Despite deliberately making time to speak with nurses face‐to‐face, participants were under significant time pressure to complete daily tasks and often found it challenging to find time to create civility within a given shift. Compounded by a reported lack of managerial communication regarding changes within the organisation, as well as micromanaging, intimidating and condescending behaviours, participants faced barriers in communicating with nurses in their teams. The following quote is an excerpt from the researcher’s memo:

Memo 23 January 2023

Hannah shared her opinion regarding how she equated respect with communicating clearly with her managers, and that this did not need to be shown through big gestures but through small acts. She spoke passionately and I could hear that this was an issue close to her heart. I think these small “acts” of respect will likely impact the developing theory.

### 7.3. Teach

Following *communicate,* the third element of the ACTS theory was *teach. Teach* portrays the process of teaching nurses to resolve workplace incivility together as a team and the long‐term implications of cultivating a culture of civility. As opposed to explicitly facilitating conflict management between nurses, as represented in the previous element, *communicate*, *teach* builds upon the knowledge regarding conflict resolution from conversations from *communicate* by encouraging nurses to speak up against workplace incivility together as a team in a group setting.

This subtle form of both teaching and encouragement facilitated broader workplace incivility management within nursing care teams, allowing participants to empower nurses to manage workplace incivility independently, thereby creating civility in the practice setting. For example, Hannah recounted an experience where nurses in her team began addressing workplace incivility with one particular nurse after she had initially spoken to the nurse.

I sort of said … “Oh, come on, [name], you’ve got to do that as part of your role.” But I found that other staff members would then pull her up on it. Like, once they had seen me pull her up, it gave them, not power, but then they started pulling her up on stuff and then because her own peers had been saying, “Oh, this isn’t good enough” … she started to change.

In contrast, many participants also described learning from incidents of poorly managed workplace incivility in their workplaces by ensuring that they avoided harmful practices that further exacerbated a poor working culture. A lack of strong managerial leadership in addressing uncivil behaviours therefore made it increasingly challenging for participants to create civility due to the absence of role‐modelling behaviours around them. Jane commented on this barrier:

[Leadership] was very limited, really limited. And there wasn’t a lot of support in helping me … there weren’t many good leaders available to even sort of use them as mentors, really, so it was really hard.

### 7.4. Support

Following *teach,* the final letter of the ACTS acronym was *support.* This element refers to the broader help or support participants provided to their teams in upholding and sustaining a culture of civility. Like the previous element, *support* involves improving the working culture but with a focus on strengthening and supporting nurses to effectively fulfil their professional roles. The current element builds upon the team approach to conflict management outlined in the previous element *teach* by describing the positive practices of participants that promoted and maintained civility across the team outside of the sphere of workplace incivility management.

*Support* consists of three distinct practices: involving nurses in decision‐making, maintaining professional and personal boundaries and focusing on the delivery of quality patient care. Many participants described seeking out nurses’ professional opinion regarding clinical and organisational goals or decisions regardless of seniority or skill, as reflected by Hannah’s statement:

I see us all working as a team … and with any decision, I’ll always involve my ANUMs [associate nurse unit managers] and the team … I’ll ask and get other people’s opinions … so I trust my staff … and I support them.

Another critical form of leader support was encouraging nurses in the team to focus on delivering quality nursing care. Part of this involved concentrating on attaining workplace goals and attending to the needs of patients, residents and their families, sometimes placing them above their own. Refocusing on delivering quality patient or resident care help nurses shift their focus from workplace incivility to what their work really meant to them. As Lucy commented*: You know you’re not there for your own good. You’re there for the ward’s good or the patient’s good.”* Having healthy boundaries between work and personal life were also important in ensuring nurses supported each other in the team, although these could be challenging to maintain, as Sarah described:

As we enter into our workplace at the door, we are leaving everything behind. What’s happening with our personal life and personal stress … So, at [the] workplace, we are a team and it’s the residents’ home and we all have to support each other.

Despite implementing these practices, part of supporting nurses in creating civility also involved escalating uncivil behaviours demonstrated by a nurse within their team to higher management. Georgia further explained:

We have a process in place … you give them [nurse displaying uncivil behaviours] time to demonstrate change and if you are continuously witnessing the same behaviour, well, then that’s really a catalyst for you to escalate to the next level.

## 8. Discussion

The findings of this study provide new insight into the experiences of nurse leaders in promoting and maintaining civility in nursing care teams in Australian regional and rural health and aged care settings. While previous research has examined the experience of nurses in fostering civility in the academic and practice settings [26, 27], this study presents new findings through the lens of nurse leadership practices in the form of the substantive theory *ACTS Theory for Creating Civility in Nursing*. By acknowledging the contextual factors of creating civility in nursing care teams, this study sheds new light on the role of the nurse leader and the surrounding factors and barriers that influence their practices in the clinical setting.

### 8.1. Acknowledge

By being aware of their emotions through reflective practice, participants were able to respond to workplace incivility more effectively, thereby fulfilling their responsibilities as leaders. Previous research indicates that mindfulness has a positive effect on promoting nurses’ feelings of being valued, resulting in improved emotional, physical and spiritual health when experiencing workplace incivility [28]. Research by Huseynova and İslamoğlu [29] found that mindfulness positively impacts nurses’ ability to address workplace incivility in the practice setting, while nurses who display uncivil behaviours are less likely to be emotionally intelligent [30, 31]. Watson [32] adds that caring for others requires “an evolved and continually evolving emotional … intelligence, consciousness, and intentionality and level of sensitivity and efficacy, followed by a continuing lifelong process and journey to self‐growth and awareness” (p. 23). Through this process, the morally courageous leader not only admits their mistakes but places the needs of others above their own [33], thereby ensuring the wellbeing of nurses in their team [34]. The positive impact of mindfulness and fostering of emotional intelligence in developing moral courage to create civility therefore requires further research.

Participants in this study often described that their managers sometimes spoke to them in a way that depreciated and underestimated their knowledge and skills as a leader, with these statements often perceived as a form of workplace incivility. Consistent with the literature, this phenomenon reflects the well‐known saying “nurses eat their young” [12, 35]. A study conducted in a rural health setting in Australia by Hawkins et al. [8] found that 25% of nurses experienced workplace incivility from their colleagues, followed second by their nurse manager. Research also suggests that nurses are less committed to their organisation, experience poorer job satisfaction and are more likely to leave the profession when they experience workplace incivility from their manager [36, 37]. Inappropriate managerial behaviours were therefore a common barrier reported by participants in this study, requiring further research.

### 8.2. Communicate

In the current study, speaking directly to nurses affected by workplace incivility in a compassionate manner was a core component of creating civility. According to Hopkinson et al. [38], effective communication involves responding to staff emotions, actively listening to staff concerns, using positive body language and providing routine feedback. Wagner et al. [39] suggests listening to nurses’ needs by devoting time and space for their thoughts and concerns to be heard. Despite the scarcity of literature exploring the role of communication in creating civility, one study by Hartung and Miller [40] found that nurses were more satisfied with their work, able to complete their daily duties more efficiently and more likely to stay within their organisation when rural nurse managers communicated positively with them. Research by Abrams et al. [41] and Kunie et al. [42] also found that motivational language, that is, providing encouragement, explaining rules and managing staff in an empathic manner [43], improved nurses’ satisfaction towards work.

Together with positive communication and motivational language, compassion was central to facilitating participants’ face‐to‐face conversations with nurses in their teams regarding workplace incivility. Compassion refers to proactively seeking understanding of the needs of others [44]. In this study, participants described adopting an empathic approach to speaking with nursing staff during face‐to‐face conversations. However, according to the literature [44], compassion involves adopting a selfless position to the recipient, as well as a positive stance to resolving conflict. As the cornerstone of nursing codes of conduct worldwide [3], further research is needed to explore the role of compassion in creating civility in nursing teams.

Many of the participants in this study faced barriers in creating civility due to poor managerial communication within their organisation. Research indicates that a breakdown in communication directly impacts the quality of the relationships held between nurse leaders and their managers, which has a negative impact on job satisfaction [45–47]. Doleman et al. [48] found that support is needed to improve this line of communication, given that nurse unit managers are unsatisfied with the communication they receive from their superiors. Greater attention to understanding how communication can be optimised between nurse leaders and their managers is therefore needed moving forward.

### 8.3. Teach

The findings of this study also indicate that participants encouraged nurses to speak openly about workplace incivility as it occurred within the nursing care team throughout a given shift. These findings align with a scoping review conducted by Ota et al. [14], where nurse leaders reinforced workplace incivility management amongst staff [16, 27]. According to Musitia et al. [49], teaching nurses how to be aware of their own behaviour and the impact of their behaviours on others is essential to changing behavioural norms and resolving workplace incivility in the practice setting [49]. However, further research is needed to gain a greater understanding of facilitating workplace incivility resolution in nursing care teams.

### 8.4. Support

In this study, participants lastly reported respecting nurses’ autonomy by involving them in decision‐making, regardless of seniority or skillset, and focusing on delivering patient care to create civility in nursing care teams. Including nurses in decision‐making and promoting teamwork has been found to promote civility in nursing care teams [13, 14]. According to Gittell [50], shared goals, knowledge and mutual respect are important factors in building effective nursing teams. Support through shared goals is a valuable practice reflected in the wider civility literature [50–52]. A study by Mann et al. [52] found that nurses who had participated in a civility intervention in a labour and birth unit developed a greater sense of teamwork and culture of respect and increased feelings of commitment towards their workplace. This is supported by research by Liu et al. [53] and Clark [54], where creating civility not only improved team performance but also prevented workplace incivility from occurring.

Study participants further described creating civility by maintaining clear boundaries between their personal and professional life and encouraging nurses in their team to do the same. The literature surrounding this topic indicates that, while work and personal life were previously largely segregated, there has been a more recent move toward integrating one’s own “self” into work [55]. Given the changes in social interaction arising from the COVID‐19 pandemic [56], as well as the increase in dual‐income families and working mothers [57], managing the balance and complexity within one’s working and personal life has become increasingly challenging for nurses. The study findings demonstrate that an understanding of balancing these boundaries is a crucial part of creating civility within nursing care teams through nursing leadership.

Lastly, participants described the process of escalating issues to their managers when nurses within their teams continued to demonstrate uncivil behaviours. However, prior to this, participants ensured that they had spoken about the potential consequences to nurses, such as deployment to a different unit or ward for a designated period. This process also included having a meeting with staff from the people and culture department with their organisations to discuss deployment to another unit or ward. Managing ongoing incidents of repeated workplace incivility was incredibly challenging and time‐consuming for participants, indicating that solutions aimed at alleviating this pressure and stress to nurses in leadership positions are needed. The ACTS theory therefore provides nurse leaders with a practical framework for managing workplace incivility. By reflecting on and implementing the practices associated with each element (*acknowledge, communicate, teach* and *support*), they can foster meaningful civility development within nursing care teams.

## 9. Recommendations

The findings of the current study reflect a need to understand the responsibilities and expectations of nurses working in leadership roles to create civility. Education regarding both the management of workplace incivility and the creation of civility should first be incorporated into the undergraduate nursing curriculum. A curriculum that includes virtual reality simulations reflecting incidents of workplace incivility has been found to effectively increase nursing students’ awareness of civility [58]. Online learning modules have also been beneficial in improving students’ self‐efficacy in defining, detecting and addressing workplace incivility in practice [59].

Given that many experienced nurses are retiring or leaving the profession, support for novice nurses stepping up into leadership roles is needed. While no literature could be sourced related to civility, Hemann [60] found that there was a significant improvement in the leadership skills of nurses who had participated in a preceptorship programme. Mentorship programmes have also been found to promote peer support and professional growth [61, 62], which can be further harnessed to gain skills in creating civility.

To address gaps in knowledge and strengthen trust between nurses and their workplaces, ongoing education to improve the skills of nurse leaders in creating civility in nursing teams is recommended [63]. Healthcare organisations concerned about the ongoing impact of workplace incivility should first consider implementing professional accountability programmes. Professional accountability programmes involve recognising both negative and positive behaviours in staff through informal and formal feedback and commonly include training, tools and resources regarding how to address unprofessional behaviours, including workplace incivility, through speaking‐up techniques [64, 65]. It is also recommended that healthcare organisations invest in training aimed at improving nurse leaders’ communication and leadership skills. Cognitive rehearsal training has been proven to effectively strengthen nurses’ communication skills in addressing workplace incivility [66–68]. Education about role‐modelling and servant leadership has also proven effective in initiating conversations regarding workplace incivility within nursing care teams [69, 70] by teaching nurse leaders how to display and facilitate compassion, resulting in more ethical working environments [71].

## 10. Limitations

A small sample size of 11 participants may limit the generalisability of the findings. Due to all participants being female, the sample also did not include representation of the 12% of male nurses reflected in the current Australian nursing population [72]. Data were carefully scrutinised to differentiate between creating civility and the absence of workplace incivility, as these two phenomena were not mutually exclusive, by consciously creating separate categories to establish conceptual boundaries. Reporting, recall and sampling bias were minimised by undertaking routine reflexive practices such as ongoing memoing and routine discussions with the researcher team. In doing so, the researcher identified contradicting categories and narratives amongst the data and her own assumptions and biases and became more aware of her rationale for seeking out participants from specific nursing specialities.

## 11. Conclusions

The constructivist grounded theory developed from this study has highlighted the essential role nurse leaders play in creating civility and the contextual barriers that arise when managing this workforce issue. The practices of creating civility, which have been conceptualised across the ACTS theory, provide a poignant reflection of nurse leaders’ experiences and serve as an empowering guide for leaders within the nursing profession to promote and maintain civility for the wellbeing of all nurses.

## Author Contributions

Conceptualization, methodology, software, formal analysis, investigation, data curation, writing–original draft, and visualization: Marianne Ota. Validation, formal analysis, resources, writing–review and editing, and supervision: Julia Gilbert. Conceptualization, writing–review and editing, and supervision: Louisa Lam. Conceptualization, methodology, writing–review and editing, and supervision: Danny Hills.

## Funding

The authors received no funding to conduct this research. Open access publishing facilitated by Federation University Australia, as part of the Wiley ‐ Federation University Australia agreement via the Council of Australasian University Librarians.

## Conflicts of Interest

The authors declare no conflicts of interest.

## Data Availability

The data that support the findings of this study are available from the corresponding author upon reasonable request.
